# RAMAN spectroscopy imaging improves the diagnosis of papillary thyroid carcinoma

**DOI:** 10.1038/srep35117

**Published:** 2016-10-11

**Authors:** Julietta V. Rau, Valerio Graziani, Marco Fosca, Chiara Taffon, Massimiliano Rocchia, Pierfilippo Crucitti, Paolo Pozzilli, Andrea Onetti Muda, Marco Caricato, Anna Crescenzi

**Affiliations:** 1 Istituto di Struttura della Materia (ISM-CNR), via del Fosso del Cavaliere 100, 00133 Roma, Italy; 2 Policlinico Universitario Campus Bio-medico, via Álvaro del Portillo 200, 00128 Roma, Italy; 3 Thermo Fisher Scientific, Strada Rivoltana, 20090 Rodano, Milano, Italy

## Abstract

Recent investigations strongly suggest that Raman spectroscopy (RS) can be used as a clinical tool in cancer diagnosis to improve diagnostic accuracy. In this study, we evaluated the efficiency of Raman imaging microscopy to discriminate between healthy and neoplastic thyroid tissue, by analyzing main variants of Papillary Thyroid Carcinoma (PTC), the most common type of thyroid cancer. We performed Raman imaging of large tissue areas (from 100 × 100 μm^2^ up to 1 × 1 mm^2^), collecting 38 maps containing about 9000 Raman spectra. Multivariate statistical methods, including Linear Discriminant Analysis (LDA), were applied to translate Raman spectra differences between healthy and PTC tissues into diagnostically useful information for a reliable tissue classification. Our study is the first demonstration of specific biochemical features of the PTC profile, characterized by significant presence of carotenoids with respect to the healthy tissue. Moreover, this is the first evidence of Raman spectra differentiation between classical and follicular variant of PTC, discriminated by LDA with high efficiency. The combined histological and Raman microscopy analyses allow clear-cut integration of morphological and biochemical observations, with dramatic improvement of efficiency and reliability in the differential diagnosis of neoplastic thyroid nodules, paving the way to integrative findings for tumorigenesis and novel therapeutic strategies.

Raman spectroscopy (RS) is a technique, which utilizes inelastic light scattering. It is capable of probing fundamental vibrations of biomolecules, representing a label-free optical technology tool. It is increasingly used in a rapidly expanding research area dedicated to biological tissue studies^1^,^2^,^3^. Presently, clinical applications of RS are extremely challenging, being severely limited mainly by the time-consuming spectral measurements. However, technological advances over the last decade have created innovative RS based tools, providing morphological investigation of large tissue areas coupled with high resolution, point-by-point spectral analysis of biochemical composition. Such a technological development has prompted a burst of rapidly growing, clinically-driven RS investigations, leading to several *in vivo* applications of RS in biomedicine^4^,^5^,^6^,^7^,^8^. With this regard, the aim of the present research is to illustrate the great potential of the RS imaging technology with increased acquisition speed and performances for improved non-destructive diagnosis and imaging of thyroid tissues.

Thyroid nodules are increasing in frequency with a parallel increase of thyroid cancer. Fine needle aspiration biopsy (FNA), followed by cytological assessment, is the best procedure for their management in a large proportion of cases. Of these, about 60–70% are classified cytologically as benign nodules, while 4–10% are deemed malignant. However, the remaining nodules (10–26% of all FNAs) have indeterminate morphological findings (inconclusive, equivocal, atypical features) and represent a clinical problem^9^, being the “indeterminate” category associated with a 15 to 48% prevalence of malignancy, mainly papillary thyroid carcinomas (PTC)^10^. As a matter of fact, histopathological evaluation of thyroid nodules, though currently representing the ‘gold standard’ for the diagnosis, is still hampered by significant levels of inter- and intra-observer variations, even among experts^11^. In recent years, molecular analysis of FNA biopsy from thyroid nodules has been performed in order to reduce the number of cytologically indeterminate cases, with the development of several diagnostic panels^12^. Through these advances, molecular diagnostics has improved the care of patients with thyroid nodules and cancer^13^; the cost of such procedures, however, should be reduced in order to increase their cost-effectiveness in standard medical practice. Moreover, genetic molecular analysis, even if extensive, does not provide any information about the biochemical profile of these tumors.

Aim of our work was to investigate the potential of RS imaging technology in improving the assessment of thyroid tissues and in supplying the differential diagnosis of thyroid nodules. As a potential non-destructive tool to support the histopathological evaluation, *in situ* RS analysis of the biochemical features of thyroid tissues was applied to discriminate (a) between healthy and PTC tissue and (b) between two variants of PTC (classical and follicular). Our results, obtained by combining morphological and biochemical observations on the same tissue section, may represent an additional step towards improved standardization and increased reliability in thyroid cancer diagnostics.

## Results

Raman spectroscopic study, performed using a Raman imaging microscope (RM), was carried out for nine patients, which underwent total thyroidectomy and received a diagnosis of PTC based on FNA at the Endocrinology Unit of University Campus Bio-medico of Rome (UCBM). The detailed description of sample preparation for Raman measurements is given in the Methods section. Shortly, frozen thyroid tissue sections collected on glass slides were submitted to RS investigation. Adjacent, Haematoxylin/Eosin stained tissue slides were used as a reference for the presence of healthy and neoplastic tissue areas. Multiple Raman biochemical maps for the healthy and PTC zones were taken for each tissue section. In Table 1, the experimental dataset is fully represented, showing the distribution of the tissue samples (healthy, PTC classical variant and PTC follicular variant) for each patient, numbered consecutively from 1 to 9.

### Biochemical profile study

**Figure 1:**
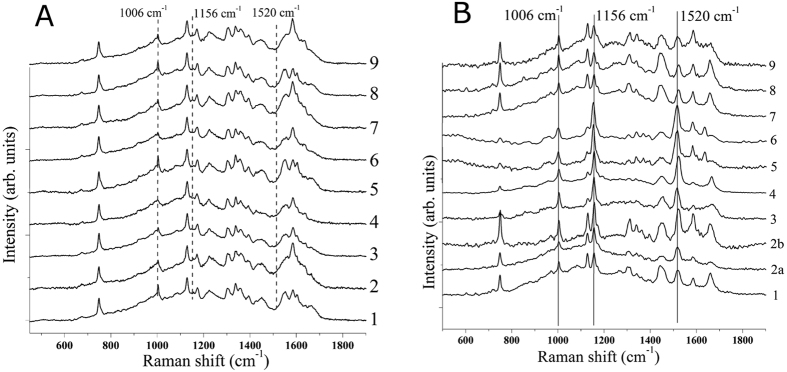
Fingerprint region: (**A**) sequence of average Raman spectra collected upon healthy thyroid tissues, (**B**) sequence of average Raman spectra collected upon PTC tissues (2(a)- follicular variant PTC and 2(b)–classical variant PTC in thyroid sample of patient 2). Spectrum numbers correspond to the thyroid case/patient number given in Table 1.

The spectra obtained by averaging the Raman biochemical maps have been classified according to the tissue type (healthy and PTC), resulting in 9 average PTC and 9 average healthy spectra, corresponding to thyroid samples from 9 patients. In Fig. 1, both sequences show the fingerprint (FP) region of spectra. In Fig. 1(A), the sequence of average Raman spectra collected upon healthy thyroid tissues is shown, whereas in Fig. 1(B), the sequence of average Raman spectra collected upon PTC tissues is presented. It should be noted that histological diagnosis evidenced the presence of zones corresponding to follicular 2(a) and to classical 2(b) PTC variants in thyroid sample of patient 2. Spectrum numbers correspond to the thyroid case numbers given in Table 1.

It should be noticed that the spectra corresponding to the same type of thyroid tissue (healthy or PTC), but belonging to different patients, are very similar to each other, demonstrating good correlation in the single case and among different cases, whereas comparison between healthy and PTC groups of spectra reveals significant differences.

An accurate assignment of the major thyroid Raman bands registered in our spectra and comparison with the literature data is given in Table 2. Available Raman literature studies regarding thyroid tissue are scarce^14^,^15^,^16^; in this study, the applied RS technique allowed us to detect for the first time some peculiar features, characteristic for thyroid tissue. The most remarkable difference between the corresponding spectra of healthy and PCT tissues consists in the presence of three intense bands at 1006, 1156 and 1520 cm^−1^ in the pathological tissue, attributable to carotenoids^14^,^17^,^18^,^19^. Indeed, the comparison between Fig. 1(A) healthy and Fig. 1(B) PTC sequences of spectra provides clear evidence that PTC tissue hosts a significant presence of carotenoids, which are otherwise just trace-like in healthy tissue. The less intense Raman band at 956 cm^−1^ (4^th^ carotenoid peak) was not distinguishable in our spectra, due to its low intensity (only about 10% of the 1156 cm^−1^ band intensity^19^). The 1006 cm^−1^ band is a mixed Raman peak, with contribution of carotenoids and phenylalanine νs (C-C) (at 1003 cm^−1^) (see Table 2).

**Figure 2:**
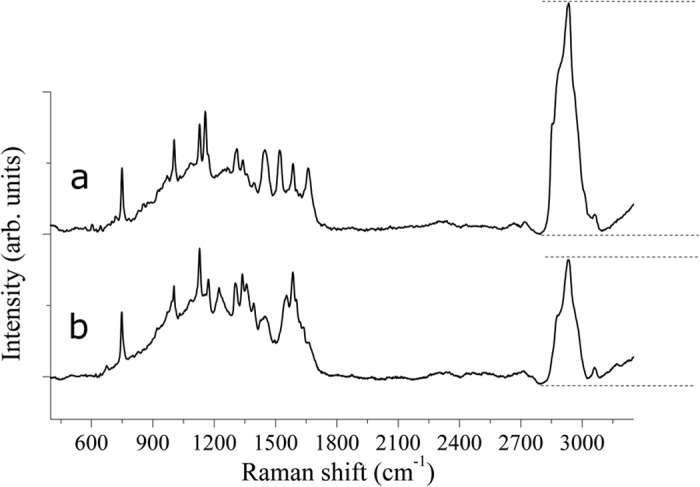
Full range Raman spectra collected upon (**a**) PTC and (**b**) healthy tissues. The shown spectra are average from the complete corresponding dataset.

In addition, the high wave number (HWN) region of spectra depicts a broad band centered at 2900 cm^−1^ (see Fig. 2 (full range spectra)), generally assigned to proteins, lipids and fatty acids vibrational modes. The ratio between this band in PTC cases and in healthy cases is approximately 2.5, therefore, indicating that another feature of PTC is a much more intense HWN band at 2900 cm^−1^.

### RS imaging

**Figure 3:**
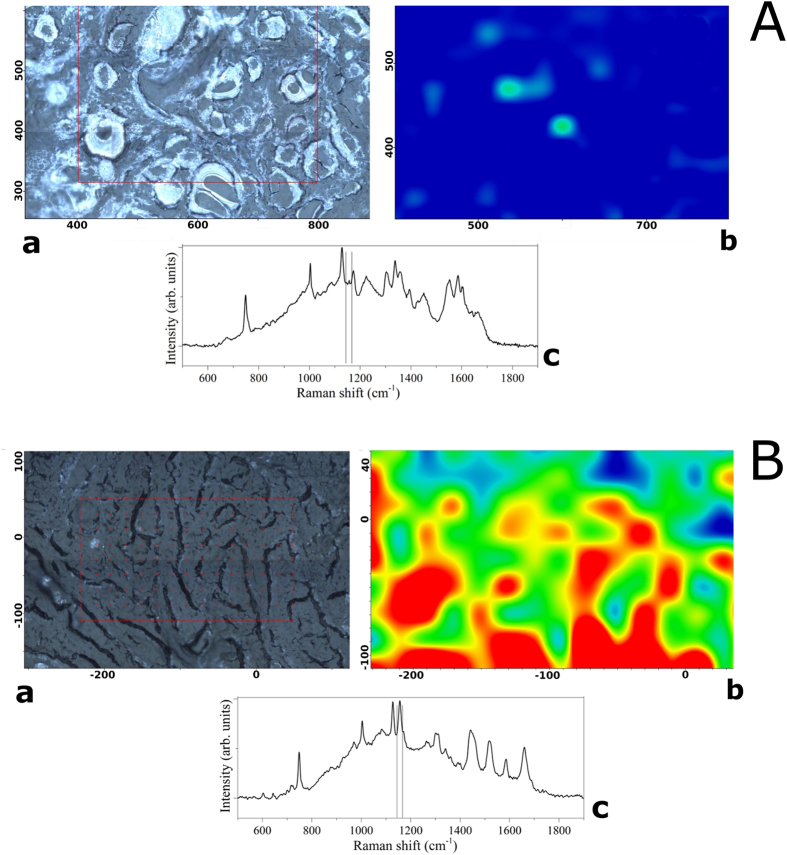
Typical example of Raman chemigram maps (1156 cm^−1^ band reference) for thyroid tissues: (**A**) healthy tissue ((a)–dark field optical image, (b)–Raman map, (c)–average reference Raman spectrum), (**B**) PTC tissue ((a)–dark field optical image, (b)–Raman map, (c)–average reference Raman spectrum). The red square on the right side (a) corresponds to the investigated tissue area shown on the left (b). The scale bars are expressed in μm.

The Raman spectra collected upon a selected area provide intrinsic biochemical information that can be used for diagnosis. By selecting specific wavelengths, Raman imaging allows one to obtain different graphical results for the maps of the two tissue typologies. An example of such maps (20 μm step size and 400 × 300 μm^2^ area) in false colors referred to the band at 1156 cm^−1^ is shown in Fig. 3. In Fig. 3(A), the results obtained for healthy tissue ((a)–dark field optical image, (b)–Raman map, (c)–average reference Raman spectrum) are presented. The healthy tissue is in blue, while minimal amounts of carotenoids are represented by the yellow-green colors. In Fig. 3(B), the PTC tissue is shown ((a)–dark field optical image, (b)–Raman map, (c)–average reference Raman spectrum). In this case, significant amounts of carotenoids in PTC tissue were detected (false colors in green-yellow-red represent the corresponding increasing intensity of carotenoids).

**Figure 4:**
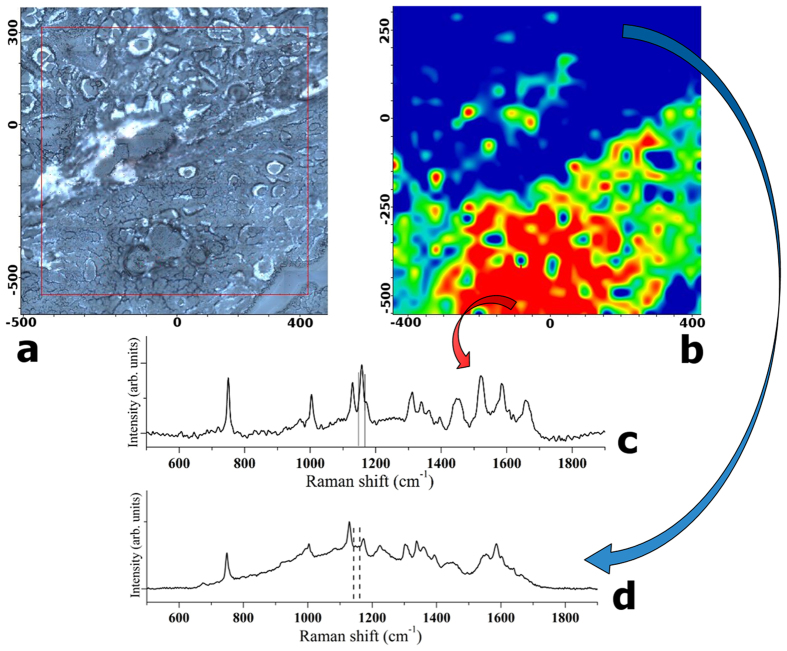
Typical example of Raman chemigram map (1156 cm^−1^ band reference) of a mixed zone of thyroid tissue (blue-healthy; red-yellow-green–PTC): ((**a**)–dark field optical image, (**b**)–Raman map, (**c**)–average reference Raman spectrum corresponding to healthy tissue, (**d**)–average reference Raman spectrum corresponding to PTC tissue). The red square on the right side (**a**) corresponds to the investigated tissue area shown on the left (**b**). The scale bars are expressed in μm.

Figure 4 depicts an area in which healthy and PTC tissues are almost intermingled; however, the applied RS technique allows one to distinguish between healthy and pathologic tissue, at least in the studied cases. This should be of capital importance when dealing with precise identification of tumor margins during excision surgery for tumors with extra-capsular extension. More data are currently being collected to address exhaustively the latter issue (using, for instance, a decreased pixel step size), and the results will be reported in the near future.

### Statistical analysis

Statistical analysis was performed on average spectra of each map, corresponding to 18 healthy tissue average spectra and 20 PTC average spectra. The FP and HWN range of Raman spectra, roughly 600 ÷ 1800 cm^−1^ and 2800 ÷ 3100 cm^−1^, were considered for statistical data treatment.

#### Discrimination between healthy and PTC tissue

The Principal Component Analysis (PCA) was performed on the matrix 38 × 1314 of the 38 average spectra in the range of 653 ÷ 1723 cm^−1^ and 2828 ÷ 3023 cm^−1^. It is reasonable to affirm that all the information about the hypothesized differences in composition is contained within the first 23 PCs, contributing to the total variability of the dataset with a larger % than the one represented by the 23^rd^ PC (the 23^rd^ PC corresponds to about 0.01%).

In order to observe whether any of the PCs have diagnostic capability (that is, the scores of the samples for that PC tend to form distinguished groups for the two tissue typologies), a two sample *t*-test was applied on the scores for each PC, after having controlled each time the validity of the assumption of equality between the variances of the two groups by Fisher’s test. The results of the *t*-test confirm that for 4 PCs (PC_1_, PC_2_, PC_5_, PC_12_) a certain separation of the mean of the healthy samples scores with respect to the one of the pathological group can be found (*p*-values of 0.028, 0.002, 0.002 and 0.050, respectively). Even if the reported *p*-values suggest a chance to use successfully this set of PCs, the check of 2D and 3D plots achievable by different combinations of such PCs shows that it is not possible. Among these combinations, for PC_1_–PC_5_, PC_2_–PC_5_ and PC_5_–PC_12_, the separation edge is simply not clear enough for diagnostic purposes. For the other cases (PC_1_–PC_2_, PC_1_–PC_12_, PC_2_–PC_12_), the test is affected by the presence of some outliers invalidating the results, when the number of observations per group is not appropriate to the degree of diversity between the distributions underlying the samples. This leads to the hypothesis that differences between tissue typologies do exist but can’t be fully revealed only by maximizing their respective variability, and truly none of the PCs by itself has diagnostic capability.

The Linear Discriminant Analysis (LDA) algorithm was first applied on the 38 × 23 matrix obtained considering the scores of the 38 samples for the first 23 PCs. Various combinations of all or part of the PCs were tried in order to find the discriminant function with the simplest mathematical solution for the classification: the best result (f_1_) was found using the PCs 1, 2, 5, 8, 10, 11, 12 and its analytical expression is:


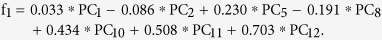


**Figure 5:**
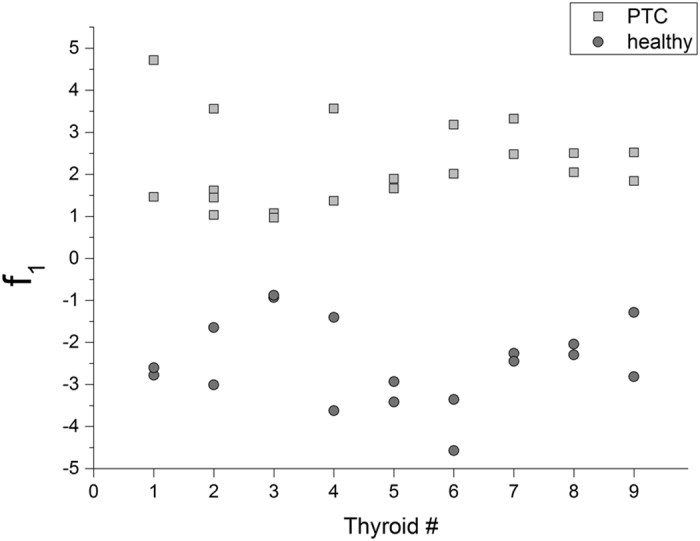
Projection of healthy and PTC samples along f_1_.

The scores for f_1_ are shown in Fig. 5. In this case, having previously verified by Fisher’s test that the variances of the sample scores on f_1_ within the two groups are not significantly different (F = 1.049; df_N_ = 19; df_D_ = 17), applying the two sample *t-*test to the same sample scores a value of t = 14.415 is obtained for 36 degrees of freedom: as a result, the two groups shown in Fig. 5 are considered significantly different at a confidence level of 0.001.

The leave-one-out cross-validation method, employed as internal tool to test the accuracy of f_1_, classified the 100% of the samples correctly. It should be also noticed that in Fig. 5 the groups are characterized by values ranging exclusively along the positive semiaxis of f_1_ (PTC tissue samples) or along the negative one (healthy tissue samples).

#### Discrimination between two PTC variants (classical and follicular)

The PCA was performed on the matrix 20 × 1314 of the 20 average spectra of PTC tissue samples in the 653 ÷ 1723 cm^−1^ and 2828 ÷ 3023 cm^−1^ ranges of spectra. The analysis of the variance values explained by each PC revealed that the first 17 components explain almost the totality of the variance (the 18^th^ PC represents <0.01%).

In this case, the values for *F*-test indicate that variances are not significantly different among the various PCs, except for PC_2_, and the *t*-test confirms that, for none of the PCs, the means of the scores for the two groups are significantly different (confidence level of 5%).

The LDA algorithm was applied also in this case and the result is the function f_2_, obtained combining the PCs 1, 4, 5, 6, 7, 8, 10, 11, 12, 15. Its analytical expression is:


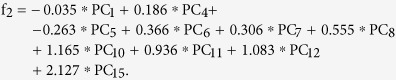


**Figure 6:**
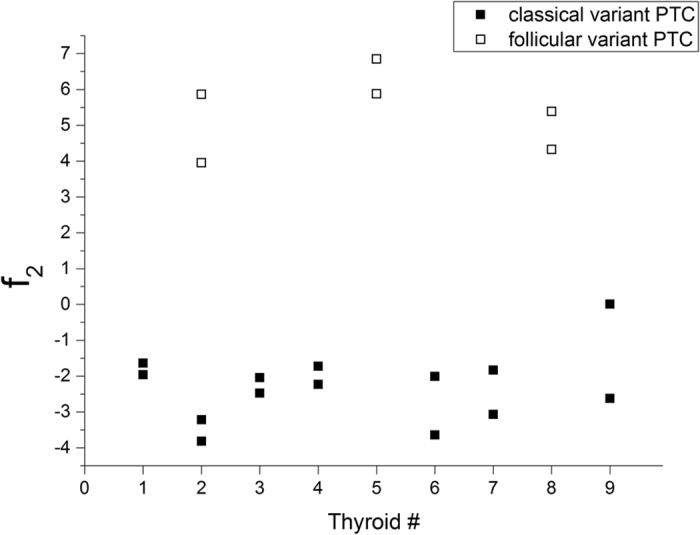
Projection of two PTC variants along f_2_.

The scores for f_2_ are shown in Fig. 6. Having previously verified by Fisher’s test that sample variances of the two groups are not significantly different (F = 0.816; df_N_ = 13; df_D_ = 5), applying the two sample *t*-test to the sample scores on f_2_, a value of t = −15.737 is obtained for 18 degrees of freedom: as a result, the two groups shown in Fig. 6 are significantly different at a confidence level of 0.001. The leave-one-out cross-validation method classified the 95.0% of the samples correctly: all the follicular variant PTC samples were attributed to the right group and, among the classical variant PTC samples, only one has been misclassified. Nevertheless, it is possible to classify the 100% of samples correctly, considering as a criterion the fact that the values of the two PTC variants are distributed along the opposite semiaxes of f_2_ (positive for follicular variant and negative for classical variant PTC).

Summarizing the results of this section, we can affirm that Raman spectroscopy is able to discriminate between healthy and PTC tissues of thyroid with 100% of sensitivity, specificity and accuracy and to discriminate between classical and follicular variants of PTC with 93% of sensitivity, 100% of specificity and 95% of accuracy, by means of the leave-one-out cross-validated LDA.

## Discussion

Our results have demonstrated the feasibility and reproducibility of RS to discriminate between normal thyroid tissue and PTC, and between classical and follicular variants of PTC, on the basis of their biochemical fingerprints. This finding is of great relevance for the development of a RS optical biopsy system to investigate thyroid tissue alterations. Based on the experimental results obtained in this work, we can attest the significant carotenoids presence in the PTC tissues with respect to the healthy tissue, in which their absence or minimal and localized presence was detected (see Figs 1, 3 and 4). To the best of our knowledge, this is the first experimental evidence of carotenoids presence in the neoplastic thyroid tissue.

Papillary thyroid carcinoma has been extensively investigated with multiplatform molecular analysis and the area of unknown genomic alteration has been reduced substantially from 25% to less than 4%^13^. Detailed study of the genomic background, however, is not sufficient to fully investigate the mechanisms that lead to neoplastic transformations, which, in turn, would lead to the identification of more accurate diagnostic, prognostic and predictive makers. For example, BRAFV600E gene mutation, currently considered as a driver of molecular alteration in classic variant PTC, has been recognized in over 70% of benign nevus without neoplastic progression^20^. Spectroscopy methods applied in clinics should provide the best possible sensitivity, specificity and accuracy in order to minimize false definitions. Ideally, a new method should be performed as an *in situ* analysis encompassing the assessment of multiple cellular constituents and allowing paired morphological and biochemical analysis. RS is among the few available methods fulfilling the above requirements.

In our study, we considered the FP and the HWN range of thyroid Raman spectra, both presenting changes while passing from healthy to PTC areas and, therefore, both important for diagnostic utility, as confirmed also by authors^6^ for cervical tissue.

RS has the ability to identify specific tumor expression molecules and molecular species involved in tumorigenesis and progression. For instance, Talari *et al.*^14^ claimed the 956, 1006, 1156–1157, 1524–1528 cm^−1^ Raman peaks as “carotenoids absent in normal tissue”. Puppels *et al.*^21^ investigated carotenoids located in human lymphocyte subpopulations and natural killer cells, evidencing a high carotenoids concentration in the CD4 + lymphocytes, and proposed to investigate the possible mechanisms behind the protective role of carotenoids against the development of cancers. The increased intensities at 1159 and 1527 cm^−1^, assigned to carotenoids have been identified also in the Raman spectra of brain tumors^14^ and neurinomas^22^. Talari *et al.*^14^ suggested that carotenoids can be used as Raman biomarkers in breast cancer pathology.

Resonance Raman is probably one of the best methods to study the properties of carotenoids in complex media, such as, for example, binding sites of biological macromolecules in living organisms^19^. In this case, i.e. when the wavelength of the excitation laser is in the range of electronic absorption band of molecules of interest, resonance Raman intensities may be enhanced up to 6 orders of magnitude, as compared to normal Raman scattering^23^. In the present work, the enhancement of carotenoid Raman bands was obtained using 532 nm laser wavelength, which lies in the range of carotenoids UV/Vis absorption region^24^.

The coupled histopathological and Raman biochemical observations performed in this work highlighted that carotenoids are mainly present in cellular areas of PTC, so that their presence seems to be related to the neoplastic thyrocytes within the tumor tissue. Our study suggests that these characteristics could be used as Raman biomarkers in the PTC pathology. However, the mechanism underlying potential oncogenic effects of carotenoids is still unknown, since very little is known about the biochemical content of neoplastic cells, especially what regards lipids, lipoproteins and lipophilic substances that are commonly lost in routinely processed histological samples. Among human tissues, different normal cells are able to utilize carotenoids, i.e. beta-carotene is reported as a local supply of vitamin A in the skin and melanocytes^25^. However, physiological mechanism for carotenoids uptake in normal thyrocytes is not reported, and our results raise the hypothesis of a carotenoid-related pathway for the PTC oncogenesis. This is just a working hypothesis, which needs accurate validation, but nevertheless underlines the presence of carotenoids in neoplastic thyrocytes, as it happens in other organs^15^,^22^,^23^.

In conclusion, we performed the RS investigation and biochemical mapping of healthy thyroid tissue and of PTC (classical and follicular variants). The obtained results demonstrate the great potential of RS to support histopathological evaluation, increasing the reliability of cancer diagnostics. On the basis of the results of multivariate statistical model, carried out by the leave-one-out cross-validated LDA, we can affirm that RS is able to discriminate between healthy and PTC tissues of thyroid with 100% of sensitivity, specificity and accuracy and to discriminate between classical and follicular variants of PTC with 93% of sensitivity, 100% of specificity and 95% of accuracy. The achieved diagnostic sensitivity, specificity and accuracy are compatible with the clinical use, both for the PTC diagnosis and in the differential diagnosis between classical and follicular variants of PTC, the latter being a significant challenging point for thyroid nodules evaluation.

Only a few literature studies report RS investigations of thyroid tissue and neoplasia^16^,^17^. The distinctive trait of our RS analysis is that for the first time it has been performed on tissue sections, combined with microscopic assessment of the very same areas. The method is highly cost-effective, being based solely on the analysis of unstained cryostatic tissue sections. Our results are likely to represent a significant advance in the imaging of thyroid tissues, leading to subsequent clinical application by improving diagnostic accuracy and reducing inter-observer variability.

This study is the first demonstration of the presence of significant amounts of carotenoids in thyroid neoplasms, suggesting that carotenoids could be used as a Raman biomarker for the PTC pathology. In this regard, combination of the histological and Raman microscopy analysis approaches may open a new way to integrative findings with wide implications for basic pathobiology, tumor classification schemes and therapeutic strategies.

## Methods

### Ethics statement

The study was approved by the Ethical Committee of the UCBM (prot. 33.15 TS ComEt CBM). Before surgical procedures, the informed consent was collected. Enrolled patients are known to the pathologist and recorded in a codified file with an anonymous ID code, which was also registered in the institutional software database of the Pathology Unit of the UCBM. All personally identifiable information was recorded in a codified file. All experiments were performed in accordance with the principle of Good Clinical Practice (GCP) and the ethical principles contained in the current version of the Declaration of Helsinki.

### Thyroid tissues

This prospective monocentric study has been approved by the Ethical Committee of the UCBM (prot. 33.15 TS ComEt CBM), and all patients gave the written informed consent. Nine patients that received a diagnosis of PTC based on FNA at the Endocrinology Unit of UCBM were enrolled for this study. These patients underwent total thyroidectomy at the Surgical Unit of the same Institution. At the time of surgery the removed specimens were immediately submitted unfixed to the Pathology Unit in an appropriately labeled container. After completion of the gross examination of the specimen by the pathologist, resection margins were marked with black ink. Pathological sampling was carried out in agreement with international guidelines for handling surgical specimens^26^. A tissue slice of about 1 × 1 × 0.3 cm^3^ was then obtained, including both healthy and neoplastic areas, avoiding surgical margins, and the slice was frozen on a metallic cold-plate inside the cryostat. A 5 μm cryostatic section was cut and stained with Haematoxylin/Eosin, in order to confirm the presence of healthy and neoplastic tissue zones, as well as the transition area between them. Additional sections were cut at 20 and 30 μm of thickness, collected on separate glass slides and stored unstained at −20 °C until the Raman evaluation. The surgical samples were subsequently fixed in buffered formalin and embedded in paraffin for permanent sectioning. Diagnosis, grading and staging were performed, in agreement with the 7°th edition of TNM^27^.

### Dataset/casuistry

A number of thyroid tissue areas was histologically identified and diagnosed as healthy or pathological (Haematoxylin/Eosin staining of frozen samples) by experienced pathologist (A.C.). By means of the RS imaging, 18 maps have been obtained from healthy and 20 from pathological areas. In Table 1, the experimental dataset is fully represented, showing the distribution of the tissue samples (healthy, PTC classical variant and PTC follicular variant) for each of the nine patients.

### Raman spectroscopic measurements

Raman spectra were recorded using a Thermo Fisher Scientific DXRxi Raman microscope at the following conditions: 532 nm laser source; 200–3400 cm^−1^ full range grating; 10× and 50× objectives; 25 μm confocal pinhole, 5 (FWHM) cm^−1^ spectral resolution. The RM instrument indicated above guarantees a fast change of experimental parameters, for better measurements procedure optimization, and does not require consumable reagents and staining treatments. As a first step, the collection of a number of mosaic images at low magnification (10×) using the RM has been carried out, providing the generic overview information on the tissue morphology and allowing one to individuate and evaluate regions of interest. After that, the region of interest was investigated collecting spectra at high magnification (50×).

Preliminary measurements were performed, in order to optimize the experimental parameters to provide a high signal-to-noise (S/N) ratio and to minimize tissue fluorescence. A 5^th^ order polynomial correction was used to compensate the tissue fluorescence. A laser power of 8 mW measured at the sample has been applied as the best compromise between the signal quality and the undesired tissue burning. The exposure time was 0.8 sec, as a suitable compromise to achieve a good spectra quality and to shorten the overall acquisition time. At least 50 exposures were averaged to obtain a high S/N ratio. Laser spot size was about 700 nm (50× objective). Various Raman maps ranging from 100 × 100 μm^2^ up to 1 × 1 mm^2^, collecting several hundreds of spectra per map, were obtained. Step size employed for small maps (i.e. 100 × 100 μm) was of about 2 μm, while for larger maps with lateral dimension ranging from several hundred of μm up to 1 mm, the average step size of about 50 μm was used. Typical collection time for each map was about 4/6 hours, both for small and large maps.

The background-subtracted Raman spectra were further normalized for the area under the curve for standardization of the tissue Raman intensities. No pre-treatment was performed on tissue samples before RS examination. To assess intra-sample variability, multiple measurements were carried out at different regions within the same sample.

### Statistical analysis

Statistical analysis was performed with a supervised approach on average spectra of each map, aiming to generate a model for classifying tissues. The same number of maps (two) from each typology of tissue present in each patient (healthy, PTC classical variant and PTC follicular variant) were employed, corresponding to 18 healthy tissue average spectra and 20 PTC average spectra. The FP and HWN range of Raman spectra, roughly 600 ÷ 1800 cm^−1^ and 2800 ÷ 3100 cm^−1^, respectively, were selected for statistical data analysis treatment. The remaining spectral range was not considered, as non meaningful from the point of view of the contained biochemical information. The collected Raman data were processed performing multivariate analysis, used for complex systems with high internal variability. Two principal statistical procedures were performed on the dataset: Principal Component Analysis and Linear Discriminant Analysis.

At first, PCA was carried out in order to reduce the initial high dimensionality of the dataset and to verify whether, in a subset of dimensions, the internal variability of the spectra can reveal on its own differences among tissue typologies that can be considered diagnostic.

LDA was applied on the same components observed in PCA to verify if differences among the typologies exist due to differences among the means of samples belonging to different tissue groups. Moreover, the implementation of the algorithm on principal components let to optimize the process of classification on the basis of a reduced number of input variables. The LDA was tested by using the leave-one-out cross validation method, in order to estimate the accuracy of our model in predicting unknown samples.

## Additional Information

**How to cite this article**: Rau, J. V. *et al.* RAMAN spectroscopy imaging improves the diagnosis of papillary thyroid carcinoma. *Sci. Rep.* **6**, 35117; doi: 10.1038/srep35117 (2016).

## Figures and Tables

**Table 1:** DATASET: Thyroid glands from 9 PTC patients.

Case	Histologic diagnosis: PTC	Number of healthy tissue maps	Number of pathological tissue maps	Total Raman maps	Total Raman spectra
1	classical	2	2	4	626
2	2a) follicular 2b) classical	2	2 + 2	6	799
3	classical	2	2	4	994
4	classical	2	2	4	669
5	follicular	2	2	4	1950
6	classical	2	2	4	2187
7	classical	2	2	4	329
8	follicular	2	2	4	713
9	classical	2	2	4	566

Total number of MAPS: 38. Total number of spectra: 8833.

**Table 2:** Peak positions and assignments of major Raman bands observed in thyroid healthy and PTC tissues.

Healthy tissue peak position (cm^−1^)	Classical PTC tissue peak position (cm^−1^)	Follicular PTC tissue peak position (cm^−1^)	Band attribution	Reference
673			tryptophan (ring breathing)	15
		717	membrane phospholipids head (C-N); adenine; lipids (CN^+^ (CH_3_)_3_)	15
748	748	748	DNA	15
		851	proline&hydroxyproline (side chain vibration); tyrosine (ring breathing and Fermi doublet); glycogen	15,16
919			proline; hydroxyproline; glycogen; lactic acid	15
		957	carotenoids; phosphates ν_s_(PO_4_^3−^); cholesterol; quinoid ring in-plane deformation	15,16
971	971		ν(C-C) wagging	15
994			C-O ribose, C-C	15,16
1003	1003	1003	phenylalanine νs (C-C)	15,17
	**1006**	**1006**	**carotenoids**	15
1031			phenylalanine (δ(C-H) and C-H in-plane bending); protein (C-N stretching); carbohydrate residues of collagen	15,16
1086	1084	1089	ν(C-C) *gauche*; ν_1_(CO_3_^2−^); ν_3_(PO_4_^3−^); ν(PO^2−^); ν(C-C) skeletal of acyl backbone in lipid (*gauche* conform.)	15,16
1128	1128	1128	proteins (C-N stretching); carbohydrates (C-O stretching); ceramides; acyl backbone in lipid (trans conform., ν(C-C))	15, 16, 17
1156	1156	1156	protein (stretching C-C and C-N)	15
**1156**	**1156**	**1156**	**carotenoids**	15
1172	1172	1172	δ(C-H), tyrosine	15
	1205		ν(C–C_6_H_5_); tryptophan; phenylalanine; adenine and tyrosine (ring breathing); amide III	16
1225			amide III (β sheet structure)	15
1234			a concerted ring mode	15
1239			amide III	15, 16
	1264	1264	lipids	15
1307	1307	1307	lipid and collagen (twisting, bending, wagging)	15
1337	1337	1337	C-H deformation (protein); amide III; glycine and proline side chain (CH_2_ wagging vibrations); adenine and guanine (ring breathing modes)	15
1360	1360	1360	tryptophan	15
1393	1393	1393	CH rocking	15
1424			lipid (CH_2_ scissoring); deoxyribose (B, Z-marker)	15
	1440/1442	1440/1442	CH, CH_2_ and CH_3_ deformation; cholesterol; triglycerides (fatty acids); lipids (CH_2_ scissoring and CH_3_ bending); collagen	15
1445			collagen (δ(CH_2_), δ(CH_3_) and CH_2_CH_3_ bending); phospholipids (δ(CH_2_), δ(CH_3_) and CH_2_CH_3_ bending); methylene (bending)	15
1450	1448/1452/1464	1448	CH_2_CH_3_, CH_2_ and CH deformation; ν(C-H); δ(CH_2_); methyl groups bending; methylene deformation; proteins (δ(CH) and δ(CH_2_))	15
1498		1498	(C-C) stretching in benzenoid ring	15
	1516/1518	1516/1518	β-carotene ν(C-C); carotenoid (C-C and conjugated C = C stretching); porphyrin ν(C = C)	14
	**1520**	**1520**	**(-C = C-) carotenoids**	15
1545			C_6_-H deformation; tryptophan	15
1552			tryptophan ν(C = C); porphyrin ν(C = C)	15
1557		1557	tryptophan; porphyrin ν(C = C); amide II (ν(CN) and δ(NH)); COO− (tyrosine, amide II)	15
1584	1584	1584	phenylalanine δ(C-C); (C-C) olefinic stretching; hydroxyproline; acetoacetate; riboflavin; lipids	15, 16, 17
1602		1602	phenylalanine δ(C-C)	15
1638	1640		water (intermolecular bending and very weak and broad ν2)	15
1660	1660	1660	ν(C-C) cis; (C-C) groups in unsaturated fatty acids; fatty acids; lipids; ceramide backbone; amide I	15
	2852	2852	ν_s_(CH_2_); lipids; fatty acids	15
2879			lipids and proteins (CH_2_ and CH)	15
	2888	2888	lipids and proteins (CH_2_ asymmetric stretching)	15
2931	2931	2931	CH_2_ asymmetric stretching	15
2936		2936	chain end CH_3_ symmetric band	15
	2960	2960	out-of-plane chain end asymmetric CH_3_ stretching	15
	3010	3010	unsaturated = CH stretching	15
